# Pulmonary Embolism Visualized via Transthoracic Echocardiogram in a Patient with Weakness and Chest Pain: A Case Report

**DOI:** 10.51894/001c.144441

**Published:** 2025-09-30

**Authors:** Zrinka Dabelic

**Affiliations:** 1 Department of Internal Medicine Henry Ford Genesys

## 75

### INTRODUCTION

Pulmonary embolism (PE) is a life-threatening condition that often requires computed tomography pulmonary angiography (CTPA) for definitive diagnosis. However, in patients with contraindications such as renal impairment, alternative imaging modalities must be considered. Transthoracic echocardiography (TTE) is a non-invasive tool that can provide indirect evidence of PE by identifying right ventricular (RV) strain and, in rare cases, direct visualization of emboli.

### CASE DESCRIPTION

An 89-year-old male with a history of atrial fibrillation, hypertension, chronic kidney disease, and prior deep vein thrombosis presented with generalized weakness, right-sided chest discomfort, and shortness of breath. He described a sudden episode of weakness while seated, accompanied by transient chest discomfort. On examination, he was tachycardic, and laboratory results were significant for elevated troponins and acute kidney injury. Electrocardiography showed sinus tachycardia with T-wave inversions in aVL, V5, and V6, raising concern for non-ST-elevation myocardial infarction (NSTEMI). The patient was started on a heparin infusion.

Given his worsening renal function, CTPA was deferred, and a TTE was performed, revealing a thrombus in the right pulmonary artery along with moderate RV dilation and severely reduced systolic function, consistent with acute PE. A ventilation-perfusion (V/Q) scan confirmed a high probability of PE. Due to his advanced age, renal dysfunction, and hemodynamic stability, percutaneous thrombectomy was not pursued, and he was managed conservatively with anticoagulation. His condition remained stable throughout hospitalization, and he was transitioned to oral apixaban before discharge.

### DISCUSSION/CONCLUSIONS

This case highlights the value of TTE in diagnosing PE, particularly in patients with contraindications to contrast-based imaging. While echocardiography is not typically the first-line diagnostic modality for PE, it can provide essential information regarding RV strain and, in rare instances, direct thrombus visualization. Early identification of PE through TTE can facilitate prompt initiation of anticoagulation, improving patient outcomes. Increased clinical awareness of echocardiography’s role in PE diagnosis may be particularly beneficial in elderly patients with renal impairment, where contrast exposure poses significant risks.

